# History of Medicine, a Neglected Subject

**Published:** 1911-08

**Authors:** Roy Blosser

**Affiliations:** Atlanta National Bank Building


### HISTORY OF MEDICINE, A NEGLECTED SUBJECT.

By Roy Blosser, M. D.

The value of the study of history is that it enables us to add to our own fund of experience by appropiating the experience of others. Emerson says: "I have no expectation that a man will read history aright who thinks that what was done in a remote age, by men whose names have resounded far, has any deeper sense than what he is doing to-day." The knowledge of an historical fact has no significance except as it is intimately related to a man's own life and work. It is for this reason that artists, musicians, and literary men, consider it of so much importance to familiarize themselves with the work of the great men who have contributed to the progress of their art. But medical men seem to have failed to realize that a knowledge of the history of their art is of any value to their own efforts in developing it. There are very few medical colleges in this country which give any systematic instruction on this subject. Anyone who will take the trouble to determine will find that our medical students go out from college with only the most meagre idea of important facts in the History of Medicine. They, probably, remember that Harvey discovered the circulation of the blood; Laveran the malarial parasite and that Pasteur is given most of the credit for bringing about the use of antiseptics in surgery. But as to the experiments which led up to these discoveries, or as to the other investigators whose work, perhaps, paved the way and made possible the final achievements, they generally have only a very dim idea or none at all.

The discovery of our present day investigators are keeping the whole world keyed up to a high pitch of expectancy, yet, if we give the matter thought, we must expect that our grandsons, who perchance will enter the medical profession, will have difficulty in naming the discoverers of the Opsonic Index, the Serum for meningitis or 606. I do not believe that the study of medical history would add any extra hardships to the work of the medical students, especially during the first and second years. Its study would be a pleasant relief from a course of study composed largely of anatomy and other foundation subjects.

Its study, at this time, would give students an increased enthusiasm for their work and a greater pride in the noble profession they are about to enter; it would give them a more serious respect and reverence for the high calling of ministering to the sick and suffering.

There is one other phase of the question that we should consider. Text books on history used in our high schools and literary colleges generally do not even mention the names of our great benefactors in the field of medicine. The writer has examined several text books on French and English History. A work on English History, rather more complete than those generally used, devoted one or two lines each to Newton, Harvey and Darwin; a similar work on French History, published in 1900, did not mention the name of Pasteur or any other medical investigator.

Should we not, as a profession, seek to have such subjects as the discovery of vaccination for small-pox, antiseptic surgery, and anesthesia given the attention which they deserve in our schools and colleges? On the subject of the discovery of antiseptic surgery and of anesthesia, Dr. Roswell Park says: "First of all, they are the two grandest medical discoveries of all time; and, secondly, they are of Anglo-Saxon origin, the one British, the other American. To the introduction of anaesthetics and antiseptics is due a complete revolution of earlier methods, complete relief of pain during surgical operations; in other words, to these two discoveries the human race owes more of the prolongation of life and relief of suffering than can ever be estimated or formulated in words.

"What an everlasting disgrace it is that, while to the great murderers of mankind, men like Napoleon in modern times and his counterpart in all times, the world over does honor, erects imposing monuments and writes volumes of encomiums and flattering histories, the men to whom the world is so vastly more indebted for all that pertains to life and comfort are scarcely ever mentioned save in medical history, while the world at large is ever ignorant of their names."

The history of achievement in America for the past century shows that our medical men are fast taking a leading place among the investigators of all countries. Of the work of our contemporaries, we cannot yet form a true estimate, but surely we will not allow the names and fame of Flint, Holmes, McDowell, Sims and Gross to be forgotten. We trust not to fail to imbue the coming generation of physicians with a just pride and veneration for those who have made possible our modern efficiency in the practice of medicine and surgery.

Atlanta National Bank Building.

